# Functions of RNA N^6^-methyladenosine modification in acute myeloid leukemia

**DOI:** 10.1186/s40364-021-00293-w

**Published:** 2021-05-17

**Authors:** Xue Zheng, Yuping Gong

**Affiliations:** Department of Hematology, West China Hospital, Sichuan University, Chengdu, Sichuan Province China

**Keywords:** N^6^-methyladenosine (m^6^A), Epigenetics, RNA methylation, Acute myeloid leukemia

## Abstract

Acute myeloid leukemia (AML) is a hematologic malignancy with an unfavorable prognosis. A better understanding of AML pathogenesis and chemotherapy resistance at the molecular level is essential for the development of new therapeutic strategies. Apart from DNA methylation and histone modification, RNA epigenetic modification, another layer of epigenetic modification, also plays a critical role in gene expression regulation. Among the more than 150 kinds of RNA epigenetic modifications, N^6^-methyladenosine (m^6^A) is the most prevalent internal mRNA modification in eukaryotes and is involved in various biological processes, such as circadian rhythms, adipogenesis, T cell homeostasis, spermatogenesis, and the heat shock response. As a reversible and dynamic modification, m^6^A is deposited on specific target RNA molecules by methyltransferases and is removed by demethylases. Moreover, m^6^A binding proteins recognize m^6^A modifications, influencing RNA splicing, stability, translation, nuclear export, and localization at the posttranscriptional level. Emerging evidence suggests that dysregulation of m^6^A modification is involved in tumorigenesis, including that of AML. In this review, we summarize the most recent advances regarding the biological functions and molecular mechanisms of m^6^A RNA methylation in normal hematopoiesis, leukemia cell proliferation, apoptosis, differentiation, therapeutic resistance, and leukemia stem cell/leukemia initiating cell (LSC/LIC) self-renewal. In addition, we discuss how m^6^A regulators are closely correlated with the clinical features of AML patients and may serve as new biomarkers and therapeutic targets for AML.

## Introduction

Acute myeloid leukemia (AML) is the most common type of acute leukemia in adults and is characterized by infiltration of malignant myeloid progenitor cells into the bone marrow, peripheral blood and other tissues, which causes uncontrolled proliferation, poor differentiation and abnormal hematopoiesis [1, 2]. Despite advances in medical treatment, less than 40% of AML patients treated with the current standard chemotherapies survive for over 5 years after diagnosis [2, 3]. Thus, a better understanding of AML pathogenesis and chemotherapy resistance at the molecular level is needed so that new therapeutic strategies can be developed. Apart from abnormal genetic changes, epigenetic regulation also plays a critical role in AML [4, 5]. Epigenetic modification, which has become a rapidly evolving field in cancer biology, involves dynamic and reversible regulation of gene expression that does not alter the DNA sequence [4, 6]. The main types of epigenetic modification are DNA methylation and histone modification [4, 6]. Importantly, some epigenetic drugs that target these modifications, such as decitabine, azacitidine and sedamine, have been approved for the treatment of hematopoietic malignancies, particularly AML, lymphoma and myelodysplastic syndromes [4, 5, 7].

Similar to DNA methylation and histone modification, RNA epigenetic modification has recently been implicated in AML initiation and progression [8]. To date, more than 150 kinds of RNA epigenetic modifications have been discovered in cellular RNA types, including messenger RNA (mRNA), transfer RNA (tRNA), ribosomal RNA (rRNA), and long noncoding RNA (lncRNA) [9, 10]. Among them, the N^6^-methyladenosine (m^6^A) RNA modification, which refers to methylation at the N^6^ position of adenosine, is the most prevalent internal mRNA modification in eukaryotes [11, 12]. Recent transcriptome-wide mapping has revealed that m^6^A sites are enriched mainly at the motif RRACH (R = G or A, H = A, C, or U) in the 3′ untranslated region (3′ UTR), in the long internal exon, and near the stop codon in mRNA [13–16]. m^6^A can be deposited on specific target RNA molecules by methyltransferases and removed by demethylases [17–20]. Moreover, m^6^A-specific binding proteins recognize and bind to the m^6^A motif of RNA, influencing RNA splicing, stability, translation, nuclear export, and localization at the posttranscriptional level [21–26]. Furthermore, reversible mRNA m^6^A modification is involved in various biological processes, such as circadian rhythms, adipogenesis, T cell homeostasis, spermatogenesis, and the heat shock response, and its dysregulation causes abnormal gene expression, leading to type 2 diabetes, cancers and other diseases [20, 27–33].

In this review, we summarize the most recent information on m^6^A RNA methylation in AML, including the biological functions and molecular mechanisms. In addition, we discuss m^6^A regulators that are closely correlated with the clinical features of AML patients and can be used as new biomarkers and therapeutic targets for AML.

### The process of m^6^A RNA modification

#### Methyltransferases (writers)

Methyltransferases, also called writers, mainly include methyltransferase-like 3 (METTL3), methyltransferase-like 14 (METTL14), and Wilm’s tumor 1-associated protein (WTAP), which are categorized as the core components of the m^6^A methyltransferase complex [17, 34]. METTL3, which binds to the methyl donor S-adenosylmethionine (SAM), was the first writer of the methyltransferase complex to be identified [35]. METTL3 and METTL14 localize to nuclear speckles and form a stable heterodimer to synergistically induce m^6^A methylation [34]. In this complex, METTL3 acts as the only catalytic subunit, and most studies have indicated that METTL14 provides structural support for METTL3 and contributes to recognition of RNA substrates without possessing independent catalytic activity [18, 36–38]. WTAP, a regulatory component that enhances the catalytic activity of the m^6^A methyltransferase complex, can interact with the METTL3-METTL14 heterodimer to support localization to nuclear speckles and recruit the complex to target mRNA [17, 38]. Since WTAP itself has no methyltransferase activity, its effect depends strictly on a functional METTL3-METTL14 heterodimer [34, 39].

Additional adaptor proteins, such as RNA-binding motif protein 15 (RBM15), vir-like m^6^A methyltransferase-associated (VIRMA, also known as KIAA1429), and zinc finger CCCH-type containing 13 (ZC3H13), have since been identified as subunits of the writer complex and contribute to catalyzing m^6^A methylation [40–42]. RBM15 and its paralog RBM15B bind the methyltransferase complex and recruit WTAP and METTL3 to their target sites in cellular mRNA and the lncRNA X-inactive specific transcript (XIST), promoting m^6^A formation and XIST-mediated gene transcriptional repression [40]. Recently, the methyltransferase subunit VIRMA was found to recruit the core components METTL3/METTL14/WTAP and mediate their preferential m^6^A deposition in the 3′ UTR and near the stop codon [43]. The methyltransferase subunit ZC3H13 plays a critical role in anchoring the complex in the nucleus to improve its catalytic activity [44].

Methyltransferase-like 16 (METTL16) is a newly discovered m^6^A methyltransferase for U6 spliceosomal small nuclear RNA [45]. It can catalyze m^6^A modification in introns or intron-exon boundaries rather than the common m^6^A sites of the METTL3-METTL14 methyltransferase complex, suggesting that METTL16 functions independently of the complex [46]. METTL16 also participates in pre-messenger RNA (pre-mRNA) splicing and maintains SAM homeostasis [45–47].

#### Demethylases (erasers)

Demethylases, also known as erasers, are proteins that remove m^6^A methylation modifications to enable reversible and dynamic regulation; these erasers include fat mass and obesity-associated protein (FTO) and AlkB homolog 5 (ALKBH5) [19, 20]. FTO was the first identified m^6^A demethylase and plays an important role in energy homeostasis and obesity development, while the m^6^A demethylase ALKBH5 is involved in spermatogenesis [19, 20, 48, 49]. The two proteins belong to the AlkB family and predominantly localize with nuclear speckles, catalyzing m^6^A demethylation on nuclear RNA in an iron(II)/α-ketoglutarate-dependent manner [19, 20].

Unlike ALKBH5, which specifically demethylates m^6^A, FTO displays demethylase activity towards both internal m^6^A and 5′ cap N^6^, 2-O-dimethyladenosine (m^6^A_m_) modifications on mRNA [50, 51]. It remains controversial whether m^6^A or m^6^A_m_ is the primary substrate of FTO. Some studies have reported that FTO has a greater impact on the expression of mRNA with m^6^A modifications than that with m^6^A_m_ modifications, while others have shown that FTO preferentially demethylates m^6^A_m_ rather than m^6^A [50–52]. However, Wei et al. found that FTO catalyzes m^6^A and m^6^A_m_ demethylation in mRNA with differential substrate preferences in the nucleus and cytoplasm [51]. They further demonstrated that FTO preferentially demethylates m^6^A in the nucleus while targeting both m^6^A and m^6^A_m_ in the cytoplasm [51]. Because the abundance of m^6^A is considerably higher than that of m^6^A_m_ in mRNA, FTO is still thought to have a more obvious impact on m^6^A demethylationthan on m^6^A_m_ demethylation even in the cytoplasm [51]. Furthermore, Su et al. confirmed that FTO mainly affects internal m^6^A demethylation rather than m^6^A_m_ demethylation in human AML cells [53].

AlkB homolog 3 (ALKBH3) is a novel m^6^A demethylase that preferentially targets m^6^A in tRNA rather than in mRNA or rRNA [54]. In addition, ALKBH3 mediates m^6^A demethylation in tRNA, facilitating efficient protein translation in vitro [54].

#### m^6^A binding proteins (readers)

The last group of m^6^A regulatory proteins, known as readers, can specifically recognize and bind to the m^6^A sites on RNA to regulate RNA fate and have diverse effects on gene expression [55]. The members of the YT521-B homology (YTH) domain family, including YTH domain family protein 1/2/3 (YTHDF1/2/3) and YTH domain-containing protein 1/2 (YTHDC1/2), are the most important m^6^A reader proteins and have conserved YTH domains that can recognize m^6^A-modified RNA [56]. Among them, YTHDC1 is the only m^6^A binding protein in the nucleus, while YTHDC2 and YTHDF1–3 localize to the cytoplasm [56, 57]. Functionally, YTHDC1 regulates splicing events and promotes the nuclear export of m^6^A-methylated mRNA to the cytoplasm by interacting with the splicing factor SRSF3 and the mRNA export receptor NXF1 [25, 26, 58]. YTHDC1 can also recognize m^6^A sites on the lncRNA XIST, which induces XIST-mediated gene transcriptional repression [40]. YTHDC2 is another reader protein with RNA helicase activity and has complex effects on m^6^A-modified transcripts [59]. YTHDC2 enhances the translation efficiency of its target RNA but then reduces target mRNA stability, ultimately decreasing target mRNA abundance [60, 61]. Among the readers in this family, YTHDF1 promotes translation and protein synthesis of its target mRNA by interacting with the translation initiation factor eIF3 and ribosomes [24]. YTHDF2 facilitates m^6^A-containing mRNA decay by selectively binding and transporting them from the translatable pool to RNA decay sites [62]. In addition, YTHDF2 recruits the CCR4-NOT deadenylase complex to promote deadenylation and degradation of methylated mRNA [63]. YTHDF3, the last cytoplasmic reader protein of the YTH domain family, can enhance mRNA translation in synergy with YTHDF1 and promote m^6^A-modified mRNA degradation in cooperation with YTHDF2 [23, 64]. Thus, YTHDF3 plays a synergistic role with YTHDF1 and YTHDF2 in RNA metabolism in the cytoplasm.

**Fig. 1:**
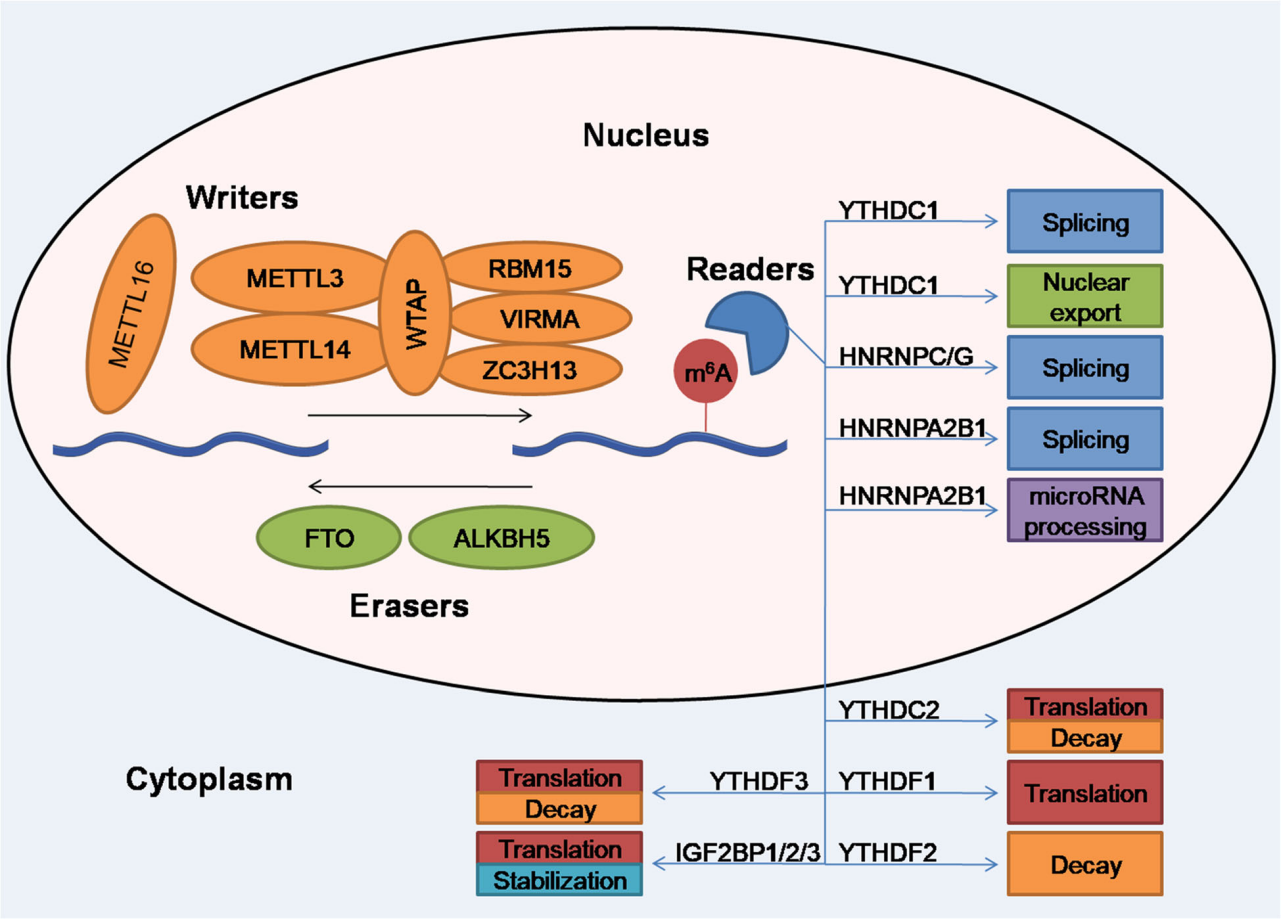
The process of m^6^A RNA modification

Apart from the YTH domain family, the heterogeneous nuclear ribonucleoprotein (HNRNP) family and insulin-like growth factor 2 mRNA-binding protein (IGF2BP) family have also been identified as m^6^A reader families [21, 65]. In the HNRNP family, m^6^A-dependent local structural alterations in RNA can increase the accessibility of methylated mRNA to HNRNPC and HNRNPG, affecting alternative splicing of target mRNA, while HNRNPA2B1 recognizes m^6^A-modified transcripts and promotes primary microRNA processing and alternative splicing [21, 22, 66]. Moreover, IGF2BP family proteins, including IGF2BP1/2/3, are a group of cytoplasmic m^6^A readers with K homology domains that recognize m^6^A-containing transcripts, thus enhancing mRNA stability and translation [65]. The functions of the major writers, erasers, and readers are summarized in Fig. 1.

m^6^A is deposited on specific target RNA molecules by the methyltransferase complex, which includes primarily METTL3, METTL14, WTAP, RBM15, VIRMA, and ZC3H13. m^6^A can also be reversibly removed by demethylases, including FTO and ALKBH5. Moreover, m^6^A-specific binding proteins, known as readers, can specifically recognize and bind to m^6^A sites on RNA, influencing RNA splicing, stability, translation, and nuclear export at the posttranscriptional level.

### The role of m^6^A RNA methylation in normal hematopoiesis and leukemogenesis

Recent studies have suggested that m^6^A RNA methylation is closely associated with normal and malignant hematopoiesis and related processes including leukemia cell proliferation, apoptosis, differentiation, therapeutic resistance, and leukemia stem cell/leukemia initiating cell (LSC/LIC) self-renewal. Herein, we summarize recent findings on the biological functions and molecular mechanisms of m^6^A regulators during normal hematopoiesis and leukemogenesis (Table 1).

**Table 1:** The roles of m^6^A regulators in normal hematopoiesis and leukemogenesis

Type	m^6^A regulators	Role in cancer	Target genes	Biological functions	Mechanism	Upstream	Reader	Refs
Leukemogenesis	METTL3	Oncogene	MYC, BCL2, PTEN	Promotes cell proliferation and colony formation, as well as inhibits differentiation and apotosis	Promotes translation of MYC, BCL2, PTEN and inhibits pAKT pathway	No study	No study	[67]
	METTL3	Oncogene	SP1	Promotes cell proliferation and inhibits differentiation	Promotes translation of SP1	CEBPZ	No study	[68]
	METTL14	Oncogene	MYB, MYC	Promotes cell proliferation, as well as inhibits differentiation and apotosis	Enhances mRNA stability and promotes translation of MYB and MYC	SPI1	No study	[69]
	WTAP	Oncogene	MYC	Promotes cell proliferation, colony formation and chemoresistance, as well as inhibits differentiation	Suppresses MYC mRNA stability	No study	No study	[70]
	WTAP	Oncogene	No study	Promotes cell proliferation, colony formation and chemoresistance, as well as inhibits differentiation	The molecular chaperone Hsp90 maintains the protein stability of WTAP	No study	No study	[71]
	FTO	Oncogene	MYC, CEBPA	Promotes cell proliferation	Enhances mRNA stability of MYC and CEBPA	No study	YTHDF2	[53]
	FTO	Oncogene	ASB2, RARA	Promotes cell proliferation and colony formation, as well as inhibits differentiation and apotosis	Suppresses mRNA stability of ASB2 and RARA	No study	No study	[72]
	FTO	Oncogene	PFKP, LDHB	Promotes aerobic glycolysis	Enhances mRNA stability of PFKP and LDHB	No study	YTHDF2	[73]
	FTO	Oncogene	MERTK, BCL2	Promotes cell proliferation and drug resistance	Enhances mRNA stability of MERTK and BCL2	No study	YTHDF2	[74]
	FTO	Oncogene	LILRB4	Promotes decitabine-induced immune evasion and makes AML cells more resistant to T cell cytotoxicity	Enhances LILRB4 mRNA stability	No study	YTHDF2	[75]
	ALKBH5	Oncogene	AXL	Promotes cell proliferation and colony formation, as well as inhibits differentiation and apotosis	Enhances AXL mRNA stability	KDM4C, MYB, Pol II	YTHDF2	[76]
	ALKBH5	Oncogene	TACC3	Promotes cell proliferation and colony formation, as well as inhibits apoptosis	Enhances TACC3 mRNA stability	No study	No study	[77]
	YTHDF2	Oncogene	TNFR2	Promotes cell proliferation and inhibits apotosis	Suppresses TNFR2 mRNA stability	No study	/	[78]
	IGF2BP1	Oncogene	ALDH1A1, HOXB4, MYB	Promotes cell proliferation, colony formation and chemoresistance, as well as inhibits differentiation	Enhances expression of ALDH1A1, HOXB4, and MYB through posttranscriptional regulation	No study	/	[79]
Normal hematopoiesis	METTL3	/	notch1a	Promotes HSPCs generation through the endothelial-to-haematopoietic transition (EHT)	Suppresses notch1a mRNA stability and inhibits Notch signaling pathway	No study	YTHDF2	[80]
	METTL3	/	No study	Maintains HSCs in a quiescent state	No study	No study	No study	[81]
	YTHDF2	/	Wnt target genes	Maintains HSCs in a quiescent state	Facilitates mRNAs decay of Wnt target genes and represses Wnt signaling	No study	/	[82]
	YTHDF2	/	Tal1	Maintains HSCs in a quiescent state	Facilitates mRNAs degradation of multiple key transcription factors (e.g., Tal1)	No study	/	[83]
	YTHDF2	/	Inflammation related genes	Sustains HSC integrity and reconstitutes multilineage hematopoiesis upon aging	Facilitates mRNAs degradation of inflammation related genes	No study	/	[84]

“/” indicates that m^6^A regulators in normal hematopoiesis did not identify a role in cancer or m^6^A binding proteins did not identify readers in their molecular mechanisms

*Refs* references

### The role of m^6^A RNA methylation in normal hematopoiesis

Normal hematopoiesis depends on hematopoietic stem cells (HSCs), which are characterized by self-renewal and multilineage differentiation capacities, as they are able to generate all blood cell types [85]. It has been reported that epigenetic regulation, including DNA methylation and histone acetylation, is involved in HSC function [86]. m^6^A RNA methylation has also been suggested to play an important role in the function of HSCs.

A recent study found that forced expression of METTL3 significantly promoted proliferation, increased colony numbers and inhibited myeloid differentiation in human hematopoietic stem/progenitor cells (HSPCs) [67]. Additionally, METTL3 mRNA is highly expressed in HSPCs but downregulated in mature differentiated myeloid cells [67]. Mechanistically, METTL3 maintains the undifferentiated state of HSPCs by increasing m^6^A modification of MYC, BCL2, and PTEN to activate translation of these proteins [67]. Another study has revealed that METTL3 maintains HSCs in a quiescent state and that conditional deletion of METTL3 in mice increases HSC numbers and initiates the cell cycle, but the mechanism still needs to be further studied [81]. It has also been reported that HSPC generation through endothelial-to-hematopoietic transition (EHT) in METTL3-deficient embryos is blocked [80]. METTL3 promotes EHT and the expansion of HSPCs in embryos by regulating different targets and pathways; for example, it decreases notch receptor 1a (notch1a) mRNA stability via YTHDF2-mediated degradation and continuous suppression of the Notch signaling pathway [80]. Similar to METTL3 expression, METTL14 expression is significantly increased in HSPCs and downregulated during myeloid differentiation [69]. Knockdown of METTL14 in normal CD34+ HSPCs substantially promotes differentiation and inhibits colony formation, but only has slight impacts on cell growth and apoptosis, implying that METTL14 plays an important role in inhibiting normal myeloid differentiation [69]. In addition, METTL14 is critical for normal hematopoiesis, as it enhances the mRNA stability and translation of the oncogenic transcription factors MYB and MYC, while METTL14 itself is negatively regulated by SPI1 [69]. However, FTO is dispensable for normal HSPCs [73]. In addition, ALKBH5 does not affect normal hematopoietic function in the steady state and plays only a very minor role in maintaining HSC self-renewal and differentiation under competitive repopulation stress [76, 77]. Similar to METTL3, many studies have shown that YTHDF2 also plays an important role in maintaining adult HSC quiescence, and YTHDF2 deletion facilitates HSC expansion without any noticeable lineage bias or leukemic potential [78, 82, 83]. Mechanistically, YTHDF2 deficiency prevents the degradation of m^6^A-modified mRNA associated with Wnt target genes and survival-related genes, and abnormal activation of Wnt signaling results in enhancement of the regenerative capacity of HSCs [82]. Another study has found that YTHDF2 suppresses HSC self-renewal by facilitating the degradation of mRNA encoding key transcription factors (e.g., Tal1) that are critical for stem cell self-renewal [83]. In addition, Mapperley and colleagues found that YTHDF2 expression is induced by inflammation and that YTHDF2 functions to downregulate multiple m^6^A-modified inflammation-related transcripts, sustain long-term HSC integrity and reconstitute multilineage hematopoiesis upon aging [84]. These findings emphasize that some m^6^A regulators may be essential for normal hematopoiesis. However, whether WTAP is involved in normal hematopoiesis remains elusive and needs further study.

### The role of m^6^A RNA methylation in LSC/LIC self-renewal

LSCs/LICs, which are characterized by properties of self-renewal and chemotherapy resistance, initiate and maintain AML and are responsible for treatment failure and disease relapse [87–89]. Therefore, novel therapeutic targets that can be exploited to selectively eliminate LSCs/LICs are needed. A recent study revealed that METTL14 is essential for the self-renewal of LSCs/LICs, as METTL14 enhances mRNA stability and promotes the translation of the oncogenic transcription factors MYB and MYC, but it is negatively regulated by SPI1 [69]. Pharmacological inhibition or knockdown of FTO also impairs LSC/LIC self-renewal capacity and decreases LSC/LIC frequency by downregulating the expression of MYC and CEBPA in an FTO/m^6^A-dependent manner [75]. In addition, ALKBH5 depletion in AML patient-derived LSCs significantly inhibits proliferation, reduces colony formation, induces apoptosis, and impairs the leukemogenic potential of the cells in immunodeficient mice [76]. ALKBH5 plays a critical role in the development and functional maintenance of LSCs by promoting the expression of the receptor tyrosine kinase (RTK) AXL via prevention of m^6^A-YTHDF2-dependent mRNA degradation [76]. Research has also shown that ALKBH5 facilitates LSC/LIC self-renewal and increases LSC/LIC frequency by stabilizing the transcript of TACC3, a prognosis-associated oncogene in multiple tumor types [77]. Furthermore*,* YTHDF2 promotes the development and maintenance of LSCs by downregulating tumor necrosis factor (TNF) receptor 2 (TNFR2) through an m^6^A-dependent pathway, and TNFR2 downregulation can protect LSCs from apoptosis [78]. The above results suggest that some of the m^6^A regulators have critical roles in promoting LSC stemness. Whether METTL3 or WTAP regulates LSC self-renewal deserves to be further studied.

### The role of m^6^A RNA methylation in leukemogenesis

A recent study showed that METTL3 depletion blocked leukemia cell growth, induced differentiation and apoptosis, and delayed leukemia development in immunodeficient recipient mice [67]. Currently, m^6^A-specific methylated RNA immunoprecipitation sequencing (MeRIP-seq or m^6^A-seq) is the most widely used method for identifying transcriptome-wide m^6^A sites [13, 14]. This method relies on m^6^A-specific antibodies to pull down m^6^A-containing RNA fragments, which are subsequently identified by high-throughput sequencing. However, its resolution is limited by the RNA fragment size (approximately 200 nucleotides). Another new method called m^6^A individual nucleotide resolution crosslinking and immunoprecipitation (miCLIP) can detect the mutations generated by UV-induced crosslinking of m^6^A-specific antibodies with methylated RNA, which can accurately identify m^6^A sites at a single-nucleotide resolution [16]. In this study, miCLIP mapping of m^6^A sites coupled with ribosome profiling indicated that METTL3 facilitates the translation of downstream targets, including MYC, BCL2, and PTEN mRNA molecules, by increasing their m^6^A levels [67]. Moreover, METTL3 leads to decreased expression of pAKT, which contributes to blockade of myeloid differentiation [67]. Another study also reported a similar phenotype and confirmed that METTL3 is critical for maintaining the undifferentiated leukemic state [68]. METTL3 is recruited by the transcription factor CEBPZ to the promoter region of its target gene, SP1, an oncogene in several cancers [68]. Binding of METTL3 to the promoter increases the m^6^A methylation level of SP1 mRNA and promotes its protein translation by relieving ribosome stalling [68]. In addition, METTL14 knockdown promotes myeloid differentiation, inhibits cell growth, induces apoptosis in vitro, significantly delays leukemia onset, and prolongs survival in immunodeficient recipient mice in vivo [69]. In addition, METTL14 is downregulated during myeloid differentiation in AML cells [69]. METTL14 promotes AML progression by positively regulating the expression of its targets, such as MYB and MYC, through enhancement of their m^6^A modification, and METTL14 transcription is negatively regulated by SPI1 [69]. Furthermore, it hasbeen reported that WTAP depletion in human AML cells induces differentiation and inhibits cell proliferation and colony formation by preventing MYC mRNA degradation through m^6^A-based posttranscriptional regulation [70]. Bansal et al. also suggested that WTAP plays an oncogenic role in AML by binding to the molecular chaperone Hsp90, which helps maintain the protein stability of WTAP by preventing its degradation via the ubiquitin-proteasome pathway [71].

Similar to writers, m^6^A erasers are also involved in leukemogenesis. Overexpression of FTO significantly enhances AML cell proliferation, increases colony numbers, and suppresses apoptosis and differentiation in vitro [72]. In addition, forced expression of FTO significantly promotes leukemic oncogene-mediated leukemogenesis in mice [72]. Collectively, these data indicate that FTO plays a critical oncogenic role by posttranscriptionally promoting the mRNA degradation of the tumor suppressors ASB2 and RARA, resulting in decreased expression of these targets [72]. In addition, ALKBH5 knockdown significantly promotes differentiation, blocks cell growth, induces apoptosis, reduces clonogenic ability in vitro and delays leukemia progression in vivo [76]. ALKBH5 knockdown promotes m^6^A methylation on the downstream target AXL, which therefore decreases AXL mRNA stability through an m^6^A-YTHDF2-dependent mechanism [76]. Moreover, downregulation of AXL caused by ALKBH5 knockdown reduces the activation of downstream kinase signaling pathways, such as the PI3K, MAPK, JAK/STAT, and NF-kB pathways, which are linked to AML chemoresistance [76, 90, 91]. ALKBH5 expression is also positively affected by KDM4C, which increases chromatin accessibility and promotes the recruitment of MYB and Pol II to the ALKBH5 promoter; this mechanism indicates the existence of a KDM4C-ALKBH5-AXL signaling axis in AML [76]. Another study has shown that ALKBH5 promotes AML progression by decreasing m^6^A modification of the target TACC3, resulting in an increase in mRNA stability rather than translation [77].

Like erasers, m^6^A readers also play essential roles in leukemogenesis. YTHDF2 knockdown in human AML cell lines decreases their proliferative capacity, increases their apoptosis rate, and impairs their engraftment ability in immunodeficient mice [78]. Mechanistically, YTHDF2 loss extends the half-life of m^6^A-modified transcripts, including those of TNFR2, whose upregulation increases the sensitivity of leukemia cells to TNF-induced apoptosis [78]. Knockdown of another m^6^A binding protein, IGF2BP1, significantly inhibits colony formation, reduces leukemia cell proliferation, enhances myeloid differentiation and delays leukemia development in immunodeficient recipient mice [79]. IGF2BP1 maintains tumorigenicity by enhancing the expression of critical transcriptional and metabolic regulators, including ALDH1A1, HOXB4, and MYB, through posttranscriptional regulation [79].

Collectively, these findings suggest that m^6^A RNA methylation is required for the development of AML, as it influences the stability and translation of different target mRNA molecules and regulates the expression of components of several pathways.

### The role of m^6^A RNA methylation in therapeutic resistance

Dysregulated expression of m^6^A regulators has been associated with therapeutic resistance. Naren et al. found that WTAP knockdown in leukemia cell lines enhances the sensitivity of the cells to the common chemotherapy drug daunorubicin by preventing MYC mRNA degradation in an m^6^A-dependent manner [70]. Another study also showed that upregulation of WTAP makes AML cells more resistant to etoposide treatment [71]. Mechanistically, the binding of WTAP to Hsp90 stabilizes the WTAP protein by preventing polyubiquitination and proteasomal degradation [71]. Moreover, FTO overexpression upregulates the expression of survival and proliferation genes in an m^6^A-dependent manner, which contributes to the induction of tyrosine kinase inhibitor (TKI) resistance in leukemia cells [74]. Upregulation of IGF2BP1 increases the resistance of leukemia cells to chemotherapeutic drugs by enhancing the expression of ALDH1A1, HOXB4, and MYB through posttranscriptional regulation [79]. These findings highlight the potential therapeutic value of targeting m^6^A regulators to combat drug resistance in AML.

### Potential clinical application of targeting m^6^A RNA methylation in AML

#### m^6^A RNA methylation as a biomarker in AML

Methyltransferase complex components (METTL3, METTL14, WTAP), demethylases (FTO, ALKBH5), and the common m^6^A binding protein YTHDF2 are all highly expressed in patients with various subtypes of AML [67, 69–72, 76–78]. Among them, METTL14 is significantly overexpressed in AML patients carrying common chromosomal translocations such as t(11q23), t(15;17), and t(8;21) [69]. Naren et al. found that there were more patients with FLT3-ITD than without FLT3-ITD in a WTAP high-expression group, while the frequency of patients with t(15;17) was reduced in the WTAP high-expression group [70]. In another study, NPM1 and FLT3-ITD mutations were also found to be closely correlated with WTAP levels [71]. In addition, overexpression of FTO has been found in AML patients with t(11q23), t(15;17), FLT3-ITD and/or NPM1 mutations [72]. ALKBH5, another m^6^A demethylase, is significantly upregulated in AML patients with a normal karyotype, inv.(16), t(11q23), and t(8;21) [76]. Moreover, high expression of WTAP, ALKBH5, or IGF2BP1 predicts a poor prognosis in AML patients [70, 76, 77, 79], while the expression of METTL3, METTL14, FTO, and YTHDF2 is not correlated with prognosis. Furthermore, WTAP protein levels are decreased in AML patients with complete remission [70]. In addition, high ALKBH5 expression is related to a significantly elevated relapse rate and is negatively associated with the duration until relapse in AML patients [76]. The above findings demonstrate that the expression levels of m^6^A regulators may be promising biomarkers for diagnosis, prognostic prediction and therapeutic response evaluation in AML patients.

#### m^6^A RNA methylation as a potential therapeutic target in AML

The above results demonstrate that knockdown of METTL14, FTO, ALKBH5 or YTHDF2 exerts a weaker inhibitory effect on normal hematopoiesis than on leukemogenesis and that m^6^A RNA methylation is important in the initiation and progression of AML, suggesting that m^6^A regulators may be potential therapeutic targets for selective eradication of leukemia cells. Currently, no specific inhibitors targeting m^6^A regulators other than FTO have been identified for the treatment of AML. R-2-hydroxyglutarate (R-2HG) is the major metabolic product of mutant isocitrate dehydrogenase 1/2 (IDH1/2) enzymes. It has been shown to suppress FTO activity, increase the global m^6^A modification level and exert antitumor effects in AML without IDH1/2 mutations by decreasing the mRNA stability of MYC/CEBPA, leading to the suppression of related pathways [53]. R-2HG also effectively suppresses aerobic glycolysis in IDH-wild-type leukemia cells by targeting the FTO/m^6^A/YTHDF2 signaling pathway to downregulate the expression of two critical glycolytic genes, PFKP and LDHB, which contributes to its antitumor effects [73]. Meclofenamic acid (MA), a nonsteroidal anti-inflammatory drug, has been identified as a highly selective inhibitor of FTO over ALKBH5 [92]. The ethyl ester form of MA (MA2) is also a specific FTO inhibitor that can inhibit tumorigenesis mediated by glioblastoma stem cells [93]. Recently, the MA derivatives FB23 and FB23–2 have been identified as two novel small molecule inhibitors of FTO that are highly selective in inhibiting FTO-mediated demethylation [52]. FB23–2 has been found to exhibit antileukemia effects in a patient-derived xenograft (PDX) AML mouse model [52]. Moreover, two potent small molecule inhibitors against FTO, CS1 and CS2, exert more effective antileukemic effects than the above reported FTO inhibitors, with minimal side effects on healthy control cells [75]. Mechanistically, targeting FTO can suppress the expression of immune checkpoint genes, especially LILRB4, in an m^6^A-dependent manner, substantially increasing AML cell sensitivity to T cell cytotoxicity and overcoming decitabine-induced immune evasion [75]. Therefore, a combination of FTO inhibitors and hypomethylating agents might exert synergistic effects in the treatment of AML. In addition, combined treatment with FTO inhibitors and nilotinib eradicates the TKI resistance phenotype and impairs leukemia cell propagation both in vitro and in vivo [74]. Importantly, combining FTO inhibitor treatment with existing therapeutic agents, such as standard chemotherapeutic agents or RTK inhibitors, enhances antitumor efficacy in leukemia patients, especially those with high FTO expression and relapsed/refractory AML [53, 74]. Thus, there is an increasing need to develop more selective and effective small molecule inhibitors targeting m^6^A regulators to treat AML.

## Conclusions

In summary, emerging research has revealed that m^6^A RNA modification influences normal hematopoiesis and leukemogenesis via regulation of the expression of different targets or pathways at the posttranscriptional level, primarily by influencing mRNA stability and translation efficiency. It is becoming clear that m^6^A modifications have broad influences on normal hematopoiesis; leukemia cell proliferation, apoptosis, differentiation, and therapeutic resistance; and LSC/LIC self-renewal. In addition, m^6^A regulators are highly expressed in AML and are related to patient survival as well as relapse rates, indicating that m^6^A regulators can serve as promising biomarkers for diagnosis, prognostic prediction and therapeutic response evaluation in AML patients. However, more large-scale and multicenter studies are needed to identify the specificity and sensitivity of m^6^A regulators as biomarkers that can contribute to individualized precision treatment of AML. Deregulation of m^6^A regulators has also been associated with drug resistance, suggesting the potential therapeutic value of targeting m^6^A regulators to combat drug resistance in AML. Moreover, FTO inhibitors such as R-2HG, FB23, FB23–2, CS1 and CS2 can suppress the progression of AML in vitro and in vivo, further indicating that m^6^A regulators may be promising therapeutic targets for AML.

One may expect that m^6^A writer and eraser genes can play opposite roles in the same cancer. However, the m^6^A regulators METTL3, METTL14, WTAP, FTO, ALKBH5, YTHDF2, and IGF2BP1 are all oncogenic in AML. Based on the current understanding of m^6^A modification in cancers, m^6^A itself is not pro-oncogenic or anti-oncogenic, but deregulation of m^6^A regulators may promote or inhibit the malignant process of AML by regulating the expression of related oncogenes or tumor suppressor genes with aberrant m^6^A levels on their transcripts. A similar phenomenon is observed for DNA methylation. For example, double knockout of DNMT3A (a DNA methyltransferase) and TET2 (a DNA demethylase) in mice can cooperatively accelerate malignancy development [94].

Compared to research on DNA methylation and histone modification, research on the role of m^6^A RNA methylation in AML is still in a relatively early stage. No specific inhibitors targeting m^6^A regulators are currently available in clinical practice. Therefore, more studies are required to further explore the mechanism and reveal the relationship between RNA m^6^A modification and AML pathogenesis. Development of more selective and effective small molecule inhibitors targeting m^6^A regulators through structural studies and large-scale chemical screening may provide not only tool compounds for future research but also novel therapeutic strategies for AML. In addition, combinations of such inhibitors and existing therapeutic agents may lead to major innovations in AML treatment in the future.

## Data Availability

Not applicable.

## Abbreviations

3′ UTR: 3′ untranslated region
ALDH1A1: Aldehyde dehydrogenase 1 family member A1
ALKBH3: AlkB homolog 3
ALKBH5: AlkB homolog 5
AML: Acute myeloid leukemia
ASB2: Ankyrin repeat and SOCS box containing 2
AXL: AXL receptor tyrosine kinase
BCL2: BCL2 apoptosis regulator
CCR4-NOT: Glucose-repressible alcohol dehydrogenase transcriptional effector
CEBPA: CCAAT enhancer binding protein alpha
CEBPZ: CCAAT enhancer binding protein zeta
DNMT3A: DNA methyltransferase 3 alpha
EHT: Endothelial-to-hematopoietic transition
eIF3: Eukaryotic translation initiation factor 3
FLT3-ITD: Fms related receptor tyrosine kinase 3-internal tandem duplication
FTO: Fat mass and obesity-associated protein
HNRNP: Heterogeneous nuclear ribonucleoprotein
HOXB4: Homeobox B4
HSC: Hematopoietic stem cell
HSPC: Hematopoietic stem/progenitor cell
IDH1/2: Isocitrate dehydrogenase 1/2
IGF2BP: Insulin-like growth factor 2 mRNA-binding protein
KDM4C: Lysine demethylase 4C
LDHB: Lactate dehydrogenase B
LILRB4: Leukocyte immunoglobulin like receptor B4
lncRNA: Long noncoding RNA
LSC/LIC: Leukemia stem/initiating cell
m^6^A: N^6^-methyladenosine
m^6^A_m_: N^6^, 2-O-dimethyladenosine
MA: Meclofenamic acid
MA2: The ethyl ester form of MA
METTL3: Methyltransferase-like 3
METTL14: Methyltransferase-like 14
METTL16: Methyltransferase-like 16
mRNA: Messenger RNA
MYB: MYB proto-oncogene, transcription factor
MYC: MYC proto-oncogene, bHLH transcription factor
notch1a: Notch receptor 1a
NPM1: Nucleophosmin 1
NXF1: Nuclear RNA export factor 1
PDX: Patient-derived xenotransplantation
PFKP: Phosphofructokinase platelet
pre-mRNA: Pre-messenger RNA
PTEN: Phosphatase and tensin homolog
RARA: Retinoic acid receptor alpha
RBM15: RNA-binding motif protein 15
rRNA: Ribosomal RNA
RTK: Receptor tyrosine kinase
R-2HG: R-2-hydroxyglutarate
SAM: *S-*adenosylmethionine
SP1: Sp1 transcription factor
SPI1: Spi-1 proto-oncogene
SRSF3: Serine and arginine rich splicing factor 3
TACC3: Transforming acidic coiled-coil containing protein 3
Tal1: T cell acute lymphocytic leukemia 1
TET2: Tet methylcytosine dioxygenase 2
TKI: Tyrosine kinase inhibitor
TNF: Tumor necrosis factor
TNFR2: TNF receptor superfamily member 1B
tRNA: Transfer RNA
VIRMA: Vir-like m^6^A methyltransferase-associated
WTAP: Wilm’s tumor 1-associated protein
XIST: X-inactive specific transcript
YTH: YT521-B homology
YTHDC1/2: YTH domain-containing protein 1/2
YTHDF1/2/3: YTH domain family protein 1/2/3
ZC3H13: Zinc finger CCCH-type containing 13
